# Children’s Adaption to Input Change Using an Abstract Syntactic Representation: Evidence From Structural Priming in Mandarin-Speaking Preschoolers

**DOI:** 10.3389/fpsyg.2019.02186

**Published:** 2019-10-01

**Authors:** Dong-Bo Hsu

**Affiliations:** Department of Chinese as a Second Language, National Taiwan Normal University, Taipei, Taiwan

**Keywords:** Mandarin, learning, preschoolers, structural priming, SVO-*ba* alternation

## Abstract

Two studies were undertaken to investigate structural priming in Mandarin-speaking three-, four- and, six-year-old children using a Mandarin-specific alternation between an SVO construction and a *ba-*construction (S*ba*OV). Structural priming occurred either as a single prime or cumulatively when a block of multiple primes with the same structure is administered. The results of Study 1 find that these preschoolers exhibited structural priming effects of similar magnitudes with the SVO-*ba* alternation across three age groups. The results of Study 2 show that they exhibited cumulative structural priming effects of similar magnitudes when there is no delay vs. a 1-day delay between their comprehension of primes and their target descriptions. The results also indicate that the participants exhibited stronger cumulative structural priming than regular structural priming. Together, these results suggest that at the age of three, children can employ an abstract syntactic representation to adapt to input changes and this adaptation operates on an implicit learning mechanism.

## Introduction

One of the essential issues in language acquisition is how children employ acquired abstract syntactic representations to produce language and how these abstract representations interact with exposure to input. Of issue is also whether this adaptation may change with age. The present study employs structural priming, which has commonly been considered a promising tool to investigate these issues (Branigan and Pickering, 2017), using the Mandarin-specific SVO-*ba* alternation illustrated in examples (1) and (2), with the aim to not only address the issues of how young children draw on an acquired syntactic representation to accommodate input statistics across different age groups but also explore a relatively lesser studied language population with regard to structural priming in children’s language.


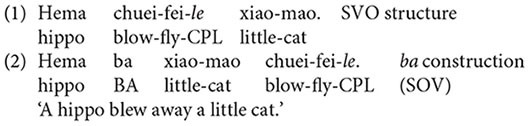


Examples (1) and (2) illustrate the simplest type of SVO-*ba* alternation in Mandarin. Although these two structures do not always alternate with each other (Huang et al., 2009), when the described event is a telic one, which has a natural endpoint after which the event cannot conceivably continue, both structures can be applied to describe the event, resulting in an SVO-*ba* alternation. The information of telicity is encoded in the verb phrase and the type of object in the sentence. The verb in both (1) and (2) is suffixed with the completion morpheme *–le* that denotes an endpoint of an action, leading to an accomplished event. In addition, the object determiner phrase (DP), *xiao-mao* ‘a little cat,’ can be exhaustively counted or measured. Once the cat is blown away, the event reaches a natural endpoint, leading to a telic event. Both of the structures have the same order of assignment of thematic roles with the agent first and the patient second, and this is one of the major properties that distinguishes it from the English transitive active-passive alternation, upon which many studies on structural priming with young children are based. Researchers (Ferreira, 2003; Huang et al., 2013) have reported that when language users comprehend sentences whose thematic role assignment does not conform to having the first NP link to the agent and the second NP to the patient, their performance is worse than for sentences that do conform to this thematic role assignment. Investigation of structural priming using this Mandarin-specific alternation allows us to shed light on how thematic role assignment may exert an impact on young children’s development of syntactic representations and how these representations may interact with input statistics.

### Structural Priming in Adult Language

With respect to production, structural priming refers to the increase in the probability of producing a recently encountered syntactic structure when there is no lexical overlap between the prime and the target. Lexical overlap between the prime and the target further strengthens the probability of producing the recently encountered syntactic structure, leading to a greater magnitude of structural priming, called the lexically boosted structural priming effect (Bock, 1986; Pickering and Branigan, 1998; Branigan et al., 2000; Chang et al., 2006; Jaeger and Snider, 2013). For example, after comprehending a prepositional dative sentence (e.g., “The pitcher sent a ball to the batter”), a speaker is likely to produce a prepositional dative sentence again when s/he encounters an event during which an agent transfers something to a recipient or beneficiary. The previously encountered structure in “The pitcher sent a ball to the batter” is known as the prime, and the subsequent content, which is examined for effects due to exposure to the prime, is referred to as the target. In her seminal work on structural priming in language production, Bock (1986) showed that once a single prime is repeated, this repetition is sufficient to lead to structural priming in subsequent sentence production; since then, subsequent work has reported that comprehending a single prime without repeating it is sufficient to induce structural priming from comprehension to production (Branigan et al., 2000; Bock et al., 2007).

Demonstrations of structural priming as learning, such as the result that structural priming persists when 10 fillers intervene between the prime and target, suggest that the mechanism that underlies structural priming is not transient activation but a learning mechanism (Bock and Griffin, 2000) that can accommodate input frequencies in language processing (Chang et al., 2006). Furthermore, demonstrations of cumulative structural priming, which occurs when structural priming involves multiple exposures of a single syntactic construction as primes, show that it produces a greater magnitude of structural priming than regular structural priming that involves a single prime (Kaschak et al., 2006; Kaschak, 2007). Kaschak et al. (2014) have reported that cumulative structural priming can be a long-lasting effect with up to 1 week between the presentation of the cumulative primes and the descriptions of the targets in a context-specific way, i.e., when the tasks between the presentation of the primes and the descriptions of the targets are identical. The authors argued that cumulative structural priming occurs (a) due to multiple exposures to the same syntactic structure that may further strengthen the syntactic representation that can be activated when only one prime is used, and (b) because it operates on an implicit learning mechanism (Bock and Griffin, 2000; Chang et al., 2006) that tracks the usage probabilities for the syntactic structures in question in a context-specific way. Jaeger and Snider (2013) argued that the tracking of the probabilities of use for syntactic structures may be a consequence of the adaptation of this learning mechanism allowing language users to minimize prediction errors in language processing. Prediction errors may occur when comprehenders experience unexpected syntactic structures, and structural priming is observed to be greater when the prediction errors are larger. The occurrence of an alignment of syntactic structures among interlocutors through structural priming, at least in part helps to reduce the subsequent prediction errors that the interlocutors may further encounter.

### Is There a Developmental Trajectory of Structural Priming in Child Language?

Most of the studies regarding structural priming in child language (Savage et al., 2003; Huttenlocher et al., 2004; Shimpi et al., 2007; Bencini and Valian, 2008; Messenger et al., 2011, 2012; Rowland et al., 2012; Peter et al., 2015) were conducted to address predictions of early-abstraction accounts (Fisher, 2002; Naigles, 2002) and usage-based accounts of language acquisition (Tomasello, 2000) regarding whether young children have adult-like abstract representations for syntax. Proponents of early-abstraction accounts, such as Fisher and colleagues (Gertner and Fisher, 2012), argued that young children and even toddlers are able to employ innately biased linking rules that are assumed to be endowed propensities between thematic roles and the number of nouns while treating the first mentioned entity as the agent to form a partial but abstract representation to guide their comprehension. As a result, children such as 2-year-olds can understand a sentence with such an arrangement of thematic roles. On the other hand, proponents of usage-based accounts (Abbot-Smith et al., 2008) denied the existence of innate biases and argued that syntactic abstraction in young children depends on the accumulation of exemplars and that how thematic roles are linked depends on the accessibilities of cues that support them in the input. Consequently, Tomasello (2003) predicted that children did not have adult-like abstract syntactic representations until the age of 3.5.

Earlier studies (Savage et al., 2003; Huttenlocher et al., 2004; Shimpi et al., 2007; Bencini and Valian, 2008) assumed that young children needed some sort of boost, such as lexical overlap between the prime and target (Savage et al., 2003) or multiple exposures of a single syntactic construction as primes (Savage et al., 2003; Huttenlocher et al., 2004; Shimpi et al., 2007; Bencini and Valian, 2008) to facilitate young children’s demonstrations of structural priming from comprehension to production, i.e., when only comprehending primes but not repeating them before proceeding to their own sentence productions. The results from these early studies suggested that children’s demonstrations of structural priming differed in terms of modalities depending on the ages they were tested, such that younger children did not demonstrate structural priming from comprehension to production up to at least the age of four, whereas older children did (Savage et al., 2003; Shimpi et al., 2007).

More recent findings regarding structural priming in young English-speaking children suggest that children as young as 3 years old can demonstrate adult-like structural priming from comprehension to production. Specifically, they exhibit structural priming after they comprehend a single prime using an active-passive alternation (Messenger et al., 2011, 2012) and dative alternation (Rowland et al., 2012; Peter et al., 2015), although a developmental trajectory seems to exist depending on which type of alternation is employed to examine structural priming in young children. Messenger et al. (2012) reported that regardless of the arrangement of thematic roles as agent-patient, theme-experiencer, or experiencer-theme in passive structures, 3- and 4-year-olds exhibited structural priming at a similar magnitude compared with adults. Messenger et al. (2011) found that 3- and 4-year-olds and adults produced full passive sentences after they comprehended a short passive sentence (e.g., “The girls are being shocked”) at a magnitude similar to that of adults. On the other hand, Rowland et al. (2012) and Peter et al. (2015) both reported a developmental trajectory in a demonstration of structural priming with the dative alternation between English-speaking 3-year-olds and their older counterparts where the magnitude of structural priming increased with age. In addition, Peter et al. (2015) reported that modulations of structural priming that are related to verbs, such as verb bias and verb overlap between the prime and target, increased with age. These more recent findings suggest preschoolers show structural priming from comprehension to production even when syntactic alternations are not concentrated in a blocked design.

English-speaking young children’s differences in magnitudes of structural priming from comprehension to production may also be attributed to the effects of different types of alternations on structural priming. Bock and Griffin (2000) have noticed that although adults can be primed with both the active-passive and dative alternations, their magnitudes of structural priming differ, possibly due to the specific structural properties that are embedded in these two types of alternation. Such a difference suggests that young children may also follow different developmental trajectories with respect to their demonstrations of structural priming effects for different alternations (see also Tomasello, 2000).

### Structural Priming as Learning in Child Language

Compared to the previously described studies that addressed the debate between early-abstraction accounts and usage-based accounts, investigations of young children’s employment of acquired abstract syntactic representations to adjust their productions to input frequencies have been sparse. As a result, it is less clear at which age young children can draw on the developed abstract syntactic representations to adapt to input statistics, as demonstrated by greater effects of cumulative structural priming over regular structural priming, and at which age their cumulative structural priming as learning is as persistent as adults’ (Kaschak et al., 2014).

Although Huttenlocher et al.’s (2004), Savage et al.’s (2006), and Kidd’s (2012) findings suggested that the underlying mechanism that preschoolers used to exhibit structural priming might involve learning, whether they would exhibit cumulative structural priming that could persist beyond the immediate context between prime presentation and target production remains unclear. As previously discussed, Huttenlocher et al. (2004) used a blocked design to induce structural priming, and their results suggest that 5-year-olds’ cumulative structural priming can persist in the immediate context, i.e., structural priming survives immediately after a blocked presentation with multiple exposures of a single syntactic construction. Moreover, Savage et al. (2006) suggested that English-speaking 4-year-olds could exhibit long-lasting structural priming with passive structures. Although their findings suggest that the structural priming effect survives up to a month, Kidd (2012) argued that Savage et al.’s (2006) results were derived from the highly atypical materials they used because they tested children only on transitive scenes where one inanimate entity acted upon another. Such materials might encourage children’s subsequent usage of passive structures, but it is unclear whether any long-lasting effect would be due to the learning mechanism itself or due to the peculiar materials. Using transitive scenes that may describe young children’s daily experiences as materials, Kidd (2012) found that English-speaking 5-year-olds exhibited cumulative structural priming for passive structures after they heard and repeated the prime, and this effect persisted into an immediate context following the priming manipulation where no further primes were administered. However, no priming effect occurred after a 1-week delay. Other studies have reported persistent and cumulative structural priming effects over several weeks with indirect-speech clauses and subordinated clauses in English-speaking 5-year-olds when multiple instances of these structures are embedded in stories (Serratrice et al., 2015; Hesketh et al., 2016). However, by definition, structural priming occurs when there is only a syntactic relation between the prime and the target, i.e., it should be independent of semantic, discoursal, or episodic structures. When primes that involve complex sentences are embedded in stories, it is likely that semantic, discoursal, or episodic structures may contribute greatly to this cumulative priming persistence.

In sum, Kidd, as well as Huttenlocher et al., suggested English-speaking 5-year-olds’ demonstrations of structural priming and cumulative structural priming may involve learning similar to that of adults (Bock and Griffin, 2000; Kaschak et al., 2014) because structural priming could survive immediately after the presentation of primes when no further primes were administered to these preschoolers. What needs to be further explored is whether young children may exhibit long-lasting effects of cumulative structural priming beyond the immediate context as adults do (Kaschak et al., 2014).

### Structural Priming in Mandarin-Speaking Preschoolers

Hsu (2014a) found that Mandarin-speaking 5-year-olds exhibited structural priming with the two structures of the SVO-*ba* alternation at a similar magnitude not only after they comprehended and produced the prime sentence (a production-to-production context), but also after they only comprehended the single prime sentence (a comprehension-to-production context). These results suggest that children employ abstract syntactic representations in both contexts (Branigan et al., 2000; Bock et al., 2007). Hsu (2014a) also found that Mandarin-speaking 5-year-olds exhibited cumulative structural priming, in which they exhibited stronger effects after they comprehended a block of multiple primes of a single syntactic structure than after they comprehended one single prime. Overall, this study suggests that 5-year-old Mandarin-speaking children can employ abstract syntactic representations (Branigan et al., 2000; Bock et al., 2007) that can be adapted to changes in input (Kaschak et al., 2006). Hsu (2014b) also reported that Mandarin-speaking 3-year-olds exhibited structural priming after they comprehended and repeated the prime sentence with this alternation.

Hsu (2018) found that Mandarin children as young as two were able to select the corresponding agent with above chance accuracy in a forced-choice pointing paradigm for both the SVO and *ba*-constructions when the verbs embedded in these constructions were novel. Furthermore, even when the subject was absent, namely, in the subjectless *ba*-construction, young children could reliably use the *ba*-marker to comprehend the agent as accurately as for the *ba*-construction with all arguments present. Overall, his findings suggest that young Mandarin-speaking children have an abstract syntactic representation for both constructions in the SVO-*ba* alternation, even though *ba*-constructions are substantially less frequent than SVO constructions. Thus, Mandarin-speaking 3-year-olds are able to exhibit structural priming. It remains to be seen how children’s structural priming develops at different ages as the structural properties of the SVO-*ba* alternation differ from the English active-passive and dative alternations, and whether children adapt to input statistics (as do adults) in a way that leads to cumulative structural priming that persists beyond the immediate context.

### Purpose of the Study

To recapitulate, it is unclear whether there is an age effect in Mandarin-speaking preschoolers’ structural priming magnitude. If so, younger children, particularly 3-year-olds, may not exhibit reliable effects as compared with their older counterparts. In this case, these young children would not exhibit greater cumulative structural priming than regular structural priming because the former relies on ‘strengthening’ already existing abstract syntactic representations. However, if the younger group, namely the 3-year-olds, have abstract syntactic representations, they should exhibit reliable structural priming effects, although it is not known whether the magnitude of their structural priming effects may differ from their older counterparts with this type of alternation. If their abstract syntactic representations adapt to the input frequencies of the primes, they should exhibit greater cumulative structural priming effects, similar to what has been found in adults with the dative alternation in English (Kaschak et al., 2006; Kaschak, 2007). Such adaptation may involve a learning mechanism that can survive beyond immediate contexts (Kaschak et al., 2014). Again, we do not know whether or not children’s adaptations to input statistics may change with age.

In sum, understanding how abstract syntax is manifested in typologically different languages, such as English and Mandarin, is important for theories of language acquisition and structural priming. If we find structural priming from comprehension to production after comprehension of one single sentence as the prime in the 3-year-old group, which is the age where studies with English-speaking children have found abstract structural priming (Messenger et al., 2012; Rowland et al., 2012; Peter et al., 2015), it would support the idea of similar abstraction processes for transitive alternations in these typologically different languages, even when the arrangements of thematic roles differ cross-linguistically. If children reliably demonstrate this structural priming effect, and if they additionally exhibit a greater cumulative structural priming effect, which persists beyond the immediate context, this would support the idea of priming as an implicit learning mechanism that keeps track of the usage probabilities of the syntactic structures in a context-specific way similar to adults. Such results would support Chang et al.’s (2006) account of structural priming which employs a dual-path model embedded in a language acquisition device that uses an error-based learning algorithm. When a speaker has formed an abstract representation of syntax, its use in the prime changes the weightings in the model and leads to structural priming. The mechanism that is subject to weight change is also responsible for learning. Thus, structural priming is a reflection of adaptation in response to changes in the input (Chang et al., 2012). The current study may provide compelling evidence for early abstraction and for the theory of structural priming as learning.

### Research Questions

Study 1 tested Mandarin-speaking 3-, 4- and 6-year-olds in a comprehension-to-production structural priming task. Study 2 tested another two groups of Mandarin-speaking 3-, 4-, and 6-year-olds using a cumulative structural priming task involving multiple exposures of a single syntactic structure as primes. One of the groups, the so-called immediate group, produced the targets immediately after the cumulative structural priming task, whereas the other group, the so-called 1-day delayed group, produced the targets with a 1-day delay. These two studies were conducted to address the following research questions:

1. Can Mandarin-speaking young children aged three, four, and six demonstrate reliable structural priming after comprehending a single prime sentence from comprehension to production?
2. Will any reliable effects differ across these age groups?
3. Do children exhibit greater cumulative structural priming than regular structural priming and will this effect differ across age groups?
4. Does a cumulative structural priming effect persist when there is 1-day delay between blocked presentation of primes and children’s descriptions of targets?

## Study 1: Structural Priming With the Svo-*Ba* Alternation

### Methods

#### Participants

Twenty-four 3-year-olds (12 boys) with an average age of 37.29 months (*SD* = 1.37, ranging from 34 to 40 months), 24 4-year-olds (12 boys) with an average age of 49.46 months (*SD* = 5.27, ranging from 42 to 54 months), and 24 6-year-olds (14 boys) with an average age of 70.42 months (*SD* = 3.13, ranging from 66 to 74 months) were recruited from 12 kindergartens in Taiwan. All participants spoke Mandarin as their dominant language, and 15 of the 3-year-olds, 16 of the 4-year-olds, and 16 of the 6-year-olds were Mandarin-Taiwanese bilinguals, who interacted with their teachers primarily in Mandarin, both in kindergarten and at home. The parents of the bilingual children reported in a background questionnaire that their children were dominant in Mandarin, but that they could interact with their grandparents in simple Taiwanese and had little difficulty understanding Taiwanese. The children had comparable socioeconomic statuses in that all of their parents were middle-class.

#### Design and Materials

This experiment employed a mixed design. Age was a between-participant variable with three levels: 3-, 4-, and 6-year-olds. Prime structure was manipulated as a within-participant variable. Each child heard both structures, the SVO construction and the *ba*-construction, with fillers interspersed between the SVO and the *ba*-constructions, i.e., a non-blocked design with fillers. Two experimental lists were prepared to counterbalance the study. List 1 began with an SVO construction (SVO-first list), and List 2 began with a *ba*-construction (*ba*-first list). Children were evenly distributed across the two lists. In addition to the experimental lists, a practice list was prepared. This practice list was administered prior to the experimental list so that the experimenter and the children could “warm up” for the later study.

Each experimental list contained 16 experimental animations and 16 filler animations. The animations were created using Adobe Flash Player 9 (Taiwan). All experimental animations denoted transitive events, which indicated a clear completion state, and could be described using an SVO structure or an S*ba*O V structure. For example, an animation that showed a hippo blowing on a cat could be described using an SVO structure as in Example (1) or a *ba* construction as in Example (2). Half of the experimental animations were used as prime animations, and half were used as target animations. A full list of the 8 target animations is shown in the Appendix, in which 4 target animations were paired with the SVO primes and 4 target animations were paired with the *ba*-construction primes. During an experimental trial, the experimenter described a prime sentence while children saw the corresponding animation, and then presented the target animation for the children to describe. The experimenter and the children took turns describing their animations. Verbs such as *scare*, *crush*, and *pull* and characters such as boy, girl, and tiger, which appeared in the animations, were lexical items that children in all age groups were expected to be familiar with (Hsu, 2014b). The lengths of the constructions were three words for the SVO construction and four words for the *ba*-construction on average, although some modifiers, such as *small* or *big*, were added to the sentences to create an event description closer to children’s utterances for those transitive events.

The sixteen filler animations allowed intransitive descriptions. Each animation involved an agent who performed a self-initiated action or an event that could be described using Mandarin’s intransitive structure. For example, one intransitive animation showed a cat standing on a chair. These fillers were interspersed with SVO-*ba* sentences in a transitive-intransitive order, where the arrangement of fillers was fixed in Lists 1 and 2.

The practice list consisted of 4 sets of practice trials including eight dative animations, which involved an agent/doer, a theme/an object, and a recipient/receiver performing a transferal event.

#### Procedure

The experimenter was allocated a room in the kindergarten to interact with the children. The children were tested individually. When they entered the room, the experimenter first asked them whether they wanted to play an animation description game with the experimenter. If they said yes, the experiment began. The experimenter first familiarized the children with the four sets of practice trials, i.e., four for the experimenter and four for the children. The experimenter told the children that they should describe the animations in order. She instructed them to carefully pay attention to her description, i.e., hearing what she described before describing their own animation. To help the children learn how to properly describe the animations, in these four trials the experimenter instructed the children to name the characters in the animation in Mandarin. For example, the experimenter might ask *zhe shi shei*? ‘Who is it?’ or *Na zhe yige you shi shei*? ‘This one, who is it?’ Then, she would ask, *Zhe yige dongzuo shi sheme*? ‘What is this action called?,’ followed by *zhege donghua li fasheng le sheme shi?* ‘What happened in the animation?’ This familiarized nearly all of the children with the task/game, so that they rarely received encouragement from the experimenter after the practice trials. During the experimental session, the experimenter and the child described all of the animations in each list in turn. For example, after the experimenter described an animation with a prime sentence, the child described their target animation with their own sentence. The experimenter subsequently described her animation with a filler sentence, and the child described their (filler) animation with their own sentence. The entire experiment ended after the experimenter and child had finished describing all of the prepared animations in the list.

#### Coding and Scoring

The children’s animation descriptions were coded according to their syntactic structures. Because the SVO and the *ba*-constructions used in the alternation are typically complex, a sentence was coded as an SVO structure if it contained a subject, a verb or verb compound, an object, and a perfective marker *–le*, in this order. It was coded as an S *ba* O V structure if it contained a subject, a *ba* marker, an object, a verb or verb compound, and a perfective marker *–le*, also in this order [see examples (1) and (2)]. Sentences that did not conform to these syntactic descriptions were coded as ‘other.’ Trials were excluded if the children repeated the experimenter’s verb to describe the target animation that immediately followed. The data were coded by a trained coder. Data from eight randomly selected children were given to another trained coder to code independently based on the previously described coding schema. Disagreements were resolved by the author. The same coding procedures were applied to all experiments in this study. Inter-coder reliability rates for the three age groups were 96% (Cohen’s *k* = 0.95, *p* < 0.001) for the 3-year-olds, 95% (Cohen’s *k* = 0.94, *p* < 0.001) for the 4-year-olds, and 96% (Cohen’s *k* = 0.95, *p* < 0.001) for the 6-year-olds.

Children also produced utterances of a code-switched *ba*-construction where they used the Southern Taiwan Min *kah*, which sounds similar to *gei* ‘give’ in Mandarin, to replace the Mandarin *ba* while they kept using the Mandarin vocabulary in the remaining sentence, as illustrated in (3).


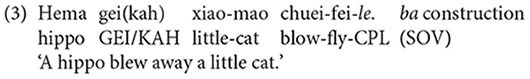


The 3-year-olds produced 15 utterances of the code-switched *ba*-construction, the 4-year-olds produced 6 utterances, and the 6-year-olds produced 2 utterances. These were also coded as *ba*-construction. The older the children are, the fewer code-switched utterances they produce. *Kah* corresponds to *ba* mostly in its grammatical usage in Mandarin; illiterate children use *gei* to substitute *kah* due to the phonological similarities between the two when they are producing *ba*-constructions in Mandarin.

### Results

The number of responses of the SVO, *ba*-constructions, and ‘other’ utterances in each priming condition across the three age groups is displayed in Table 1.

**TABLE 1:** Children’s response counts with percentages in parentheses in each priming condition across age groups.

**Condition**	**Child utterance**		
	**SVO**	**BA**	**Other**
**Age 3 priming**			
SVO	38 (40%)	28 (29%)	30 (31%)
BA	21 (22%)	48 (50%)	27 (28%)
**Age 4 priming**			
SVO	46 (48%)	27 (28%)	23 (24%)
BA	27 (28%)	50 (52%)	19 (20%)
**Age 6 priming**			
SVO	42 (44%)	30 (31%)	24 (25%)
BA	26 (27%)	43 (45%)	27 (28%)

To investigate whether (1) young Mandarin-speaking children (3-, 4-, and 6-year-olds) can exhibit structural priming and (2) whether this effect may vary across age groups, various logit mixed effect models were fitted to the data (Jaeger, 2008). All of these models were calculated using the glmer function of the *lm4* package in R (lme4: version 1.1–14). All factor labels were transformed into numerical values and centered prior to analysis to result in a mean of 0 and a range of 1. The age factor consisted of three levels, namely, 3, 4, and 6, which were later centered for statistical analysis. The reference level for this factor was 3-year-olds. This procedure minimized collinearity between variables (Baayen, 2008). Maximal models were fitted, and random slopes were simplified until the models converged (Barr et al., 2013). We report two analyses: one excludes ‘other’ responses and the other includes ‘other’ responses to determine whether the exclusion of ‘other’ responses would affect the overall results. When other responses were excluded, the analysis coded BA response as 1 and SVO response as 0. When ‘other’ responses were included, BA responses were coded as 1 while both SVO and ‘other’ responses were coded as 0. For each result, we report the log-odds coefficient for each independent variable, and its *p*-value. We also report marginal *R*^2^, namely, *R*^2^_GLMM(_*_m_*_)_, which captures variance explained by fixed factors, and conditional *R*^2^, namely, *R*^2^_GLMM(_*_c_*_)_, which captures variance explained by both fixed and random factors, as measures of the goodness fit of the models (Nakagawa and Schielzeth, 2013; Johnson, 2014; Nakagawa et al., 2017). These two types of *R*^2^ for GLMMs are implemented in the *MuMIn* package in R. These R^2^ measures evaluate the fit of models and can be compared across studies in a way similar to the standardized effect size statistics (Nakagawa and Schielzeth, 2013).

**FIGURE 1:**
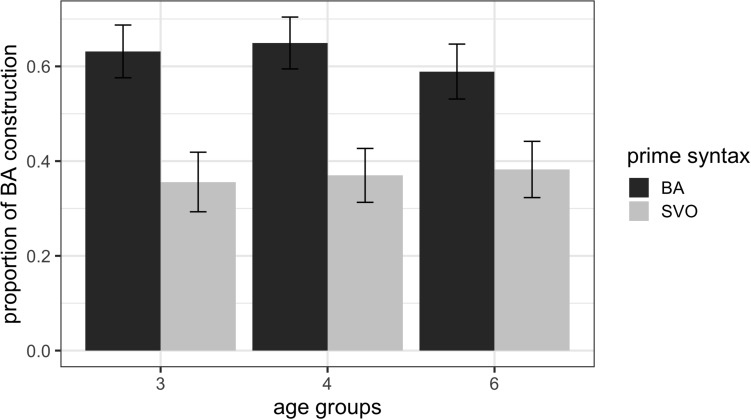
Mean proportions of *ba*-construction utterances with error bars representing standard errors of the means after SVO primes and BA primes across the three age groups.

Structural priming is demonstrated if there is a greater proportion of BA responses after BA primes than after SVO primes. Figure 1 shows the mean proportion of BA responses after BA and SVO primes across the three age groups. As shown in Figure 1 the mean proportion of BA responses was numerically greater after BA primes than after SVO primes.

We investigated whether the children’s demonstration of structural priming differed across age groups. The mixed logit model included Prime Syntax (SVO vs. BA), Bilingualism (monolingual vs. bilingual), and Age (3 vs. 4 vs. 6.) as fixed effects and treated participants and items as random variables; the model also included by-participant random slopes and by-item slopes for Prime Syntax. The results of this model that excluded ‘other’ responses are summarized in Table 2.

**TABLE 2:** Mixed logit model excluding ‘other’ responses.

**Mixed logit analysis results**				

**Predictor**	**Coefficient**	**Std. error**	**Wald Z**	***p*-value**
Intercept	–0.0496	0.2297	–0.216	0.829
Prime syntax	1.1631	0.2330	4.992	<0.001
Age	–0.0362	0.1090	–0.332	0.740
Bilingualism	–0.0186	0.2796	–0.067	0.947
Prime syntax × age	–0.1205	0.1791	–0.673	0.501
Prime syntax × bilingualism	0.2267	0.4614	0.491	0.623
Age × bilingualism	0.2403	0.2255	1.066	0.287
Prime Syntax × Age × Bilingualism	–0.0768	0.3720	–0.206	0.36

The model indicated a main effect of Prime Syntax. No other effects were significant. Thus, children showed a reliable structural priming effect that did not differ across age groups. The theoretical *R*^2^_GLMM(_*_m_*_)_ of the model was 0.0831, and the theoretical *R*^2^_GLMM(_*_c)_* was 0.2505, suggesting that both fixed and random effects explain relatively little of the variability in syntactic structures that children use. The same overall results were obtained with the model that included ‘other’ responses, except that the intercept was significant (*B* = −0.6137, *SD* = 0.2600, *z* = −2.360, *p* = 0.0183).

The results suggest that Mandarin-speaking preschoolers, including 3-, 4-, and 6-year-olds, have abstract syntactic representations, which is in line with the findings from English active-passive alternation (Messenger et al., 2011, 2012; Kidd, 2012) and dative alternation (Rowland et al., 2012; Peter et al., 2015). Overall, structural priming with the SVO-*ba* alternation in Mandarin-speaking preschoolers is not modulated by age and may occur after the preschoolers comprehended, but did not repeat, the prime. The results seem to support an early abstraction account, where no lexical overlap is necessary to obtain a structural priming effect at the age of 3.

## Study 2: Cumulative Structural Priming With the Svo-*Ba* Alternation

Study 2 investigates whether young children are able to use their abstract syntactic representations to track the exposure probabilities of the syntax they receive, leading to cumulative structural priming. Furthermore, the structural priming effect is predicted to survive beyond the immediate context between the prime presentations and target production for all age groups, similar to results from adults (Kaschak et al., 2014).

Study 2 comprises two conditions to test these predictions. One condition tests 3-, 4-, and 6-year-olds immediately after their exposure to a cumulative presentation of a single prime type (immediate condition). The other condition tests their responses 1-day after their exposure to this cumulative presentation (delayed condition).

### Methods

#### Participants

For the immediate condition, 24 3-year-olds (12 boys) with an average age of 36.46 months (*SD* = 1.64, ranging from 30 to 40 months), 24 4-year-olds (11 boys) with an average of 49.33 months (*SD* = 4.01, ranging from 42 to 54 months), and 24 6-year-olds (13 boys) with an average age of 70.75 months (*SD* = 5.80, ranging from 66 to 78 months) who did not participate in the previous experiment were recruited from 14 kindergartens in Taiwan. All participants spoke Mandarin as their dominant language, and 15 of the 3-year-olds, 16 of the 4-year-olds, and 17 of the 6-year-olds were Mandarin-Taiwanese bilinguals. For the delayed condition, an additional 24 3-year-olds (12 boys), 24 4-year-olds (11 boys), and 24 6-year-olds (12 boys) who did not participate in the previous experiments were recruited from 12 kindergartens in Taiwan. In this second group, the average age for the 3-year-olds was 38.38 months (*SD* = 2.10, ranging from 35 to 41 months), 48.96 months (*SD* = 3.58, ranging from 42 to 54 months) for the 4-year-olds, and 70.4 months (*SD* = 3.43, ranging from 66 to 78 months) for the 6-year-olds. All participants spoke Mandarin as their dominant language, and 16 of the 3-year-olds, 16 of the 4-year-olds, and 18 of the 6-year-olds were Mandarin-Taiwanese bilinguals. All of these children interacted with their teachers primarily in Mandarin, both in kindergarten and at home. The parents of the bilingual children reported in a background questionnaire that their children were dominant in Mandarin, but that they could interact with their grandparents in simple Taiwanese and that they had little difficulty understanding Taiwanese. The children had comparable socioeconomic statuses in that all of their parents were middle-class.

#### Materials

The materials are identical to those used in Study 1.

#### Procedure

##### Immediate condition

The procedure in Study 2 was similar to that used in Study 1, except that a blocked design was employed. The experimenter first administered practice trials to familiarize the children with the procedure. She described all four animations using only one dative construction, either the prepositional dative or the double-object dative, and the children then described all their four animations. If the children showed any sign of hesitation or misunderstanding, the experimenter provided them with hints or instruction. After the familiarization trials, the experimenter described all eight animations using only one of the priming structures, either the SVO construction or the *ba*-construction. Thus, prime structure was a between-participant factor, and each child would hear 8 SVO or 8 *ba*-constructions as primes. The children were instructed not to repeat what the experimenter had said, but to proceed to directly describing their own animations until the list of target animations was exhausted.

##### Delayed condition: after a 1-day intervention

All of the procedures in this experiment were identical to those in the immediate condition with the exception of the last stage. After the children were exposed to eight animations of a single structure, either the SVO construction or the *ba*-construction, they were told by the experimenter that they would continue this animation-describing game the next day. The experimenter came to the kindergarten the next day at approximately the same time and showed the eight target animations to the children who had been exposed to the eight prime animations the day before and instructed them to describe the eight target animations.

#### Coding and Scoring

The coding and scoring were identical to Study 1. All data were coded by a trained coder. Data from eight randomly selected children for each experiment were given to another trained coder to code independently based on the previously described coding schema. In the immediate condition, the reliability rate for the 3- and 4-year-olds was 95% (Cohen’s *k* = 0.94, *p* < 0.001), and for the 6-year-olds, it was 97% (Cohen’s *k* = 0.96, *p* < 0.001). In the delayed condition, the reliability rate was 96% (Cohen’s *k* = 0.95, *p* < 0.001) for the 3-year-olds, 95% (Cohen’s *k* = 0.94, *p* < 0.001) for the 4-year-olds and 96% (Cohen’s *k* = 0.95, *p* < 0.001) for the 6-year-olds.

Bilingual children again produced several utterances of the mixed *ba*-constructions: 12 utterances in the 3-year-olds, 8 utterances in the 4-year-olds, and 3 utterances in the 6-year-olds in the immediate condition; and 11 utterances in the 3-year-olds, 5 utterances in the 4-year-olds, and 4 utterances in the 6-year-olds in the 1-day delayed condition. They were again coded as *ba*-constructions.

### Results and Discussion

The number of SVO, BA and ‘other’ responses in each priming condition and in each immediacy condition across the three age groups is displayed in Table 3.

**TABLE 3:** Children’s response counts with percentages in parentheses in each priming condition and immediacy condition across age groups.

**Condition**	**Child utterance**		
	**SVO**	**BA**	**Other**

**Immediate**			

**Age 3 cumulative priming**			
SVO	49(51%)	13(14%)	34(35%)
BA	11(11%)	66(69%)	19(20%)
**Age 4 cumulative priming**			
SVO	57(59%)	7(7%)	32(33%)
BA	16(17%)	70(73%)	10(10%)
**Age 6 cumulative priming**			
SVO	59(61%)	26(27%)	11(11%)
BA	10(10%)	77(80%)	9(9%)

**1-day delay**			
**Age 3 cumulative priming**			
SVO	47(49%)	12(13%)	37(39%)
BA	11(11%)	68(71%)	17(18%)
**Age 4 cumulative priming**			
SVO	56(58%)	15(16%)	25(26%)
BA	18(19%)	71(74%)	7(7%)
**Age 6 cumulative priming**			
SVO	56(58%)	26(27%)	14(15%)
BA	10(10%)	74(77%)	12(13%)

We first investigated whether Mandarin-speaking 3-, 4-, and 6-year-olds exhibit cumulative structural priming that is greater in magnitude than the structural priming in Study 1. As in Study 1, we report one analysis that excluded ‘other’ responses and one analysis that included them.

**FIGURE 2:**
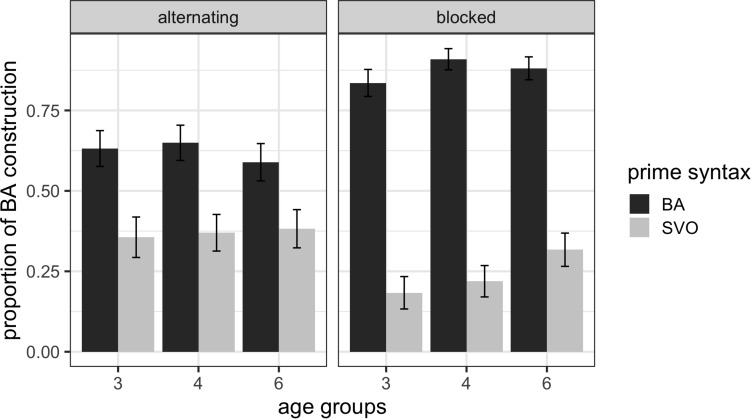
Mean proportions of *ba*-construction utterances with error bars representing standard errors of the means after SVO primes and BA primes across the three age groups in the structural priming and immediate cumulative structural priming conditions.

Figure 2 shows the mean proportion of *ba*-construction utterances produced by children in the three age groups in the structural priming in Study 1 and the immediate cumulative structural priming in Study 2.

To determine whether the children’s demonstration of structural priming and cumulative structural priming differed in magnitude across age groups, we ran a mixed logit model that included the following variables as fixed effects: (a) Age (3 vs. 4 vs. 6); (b) Prime Syntax (SVO vs. BA); (c) Prime Type (alternating, i.e., children and the experimenter took turns describing their animations in Experiment 1, vs. blocked, i.e., children received a single syntactic structure in Experiment 2); and (d) Bilingualism (monolingual vs. bilingual). Participants and Items were treated as random variables. The results of the model excluding ‘other’ responses are summarized in Table 4.

**TABLE 4:** Mixed logit model excluding ‘other’ responses: priming vs. cumulative priming.

**Mixed logit analysis results**				
**Predictor**	**Coefficient**	**Std. error**	**Wald Z**	***p*-value**
Intercept	0.2433	0.2126	1.144	0.2525
Prime syntax	2.3621	0.1994	11.847	<0.001
Age	0.0758	0.0958	0.791	0.4287
Prime type	0.5532	0.1781	3.106	0.0019
Bilingualism	0.2691	0.2277	1.182	0.2374
Prime syntax × age	–0.2172	0.1515	–1.790	0.0735
Prime syntax × prime type	2.3338	0.3798	6.145	<0.001
Age × prime type	0.2014	0.1417	1.421	0.1552
Prime syntax × bilingualism	0.6433	0.3876	1.660	0.0970
Prime type × bilingualism	0.5333	0.3828	1.393	0.1636
Age × bilingualism	0.0552	0.1785	0.309	0.7572
Prime syntax × age × prime type	–0.2409	0.3034	–0.794	0.4273
Prime syntax × age × bilingualism	–0.0091	0.3074	–0.030	0.9764
Prime syntax × prime type × bilingualism	0.6852	0.7695	0.890	0.3733
Prime type × age × bilingualism	–0.1250	0.3055	–0.409	0.6824
Prime syntax × age × prime status × bilingualism	0.2177	0.6116	0.356	0.7218

The results indicate that the main effects of Prime Syntax and Prime Type and the interaction effect of Prime Syntax and Prime Type were significant (all *p* < 0.01), whereas the remaining effects were not significant (all *p* > 0.05). The main effect of Prime Syntax shows that there was an overall priming effect with more BA responses after a BA prime than after an SVO prime. The main effect of Prime Type shows that there were more BA responses for cumulative compared to regular priming. The Prime Syntax by Prime Type interaction shows that, as predicted, children exhibited a greater magnitude of cumulative structural priming than of structural priming. The theoretical *R*^2^_GLMM(_*_m_*_)_ of the model is 0.3290, and the theoretical *R*^2^_GLMM(_*_c)_* is 0.4473. The fixed effects explain substantially more of the variability in the syntactic structures that children use than in Study 1, and the random effects seem to explain comparatively little of the variability in the syntactic structures that children use. Again the same overall results were obtained for the model with ‘other’ responses, except that the intercept (*B* = −0.4792, *SD* = 0.1939, *z* = −2.461, *p* = 0.0139), the interactions of Age and Prime Syntax (*B* = −0.2759, *SD* = 0.1266, *z* = −2.179, *p* = 0.0293) and of Age and Prime Type (*B* = 0.2823, *SD* = 0.1160, *z* = 2.433, *p* = 0.0150) were also significant. These results may occur because age was not significant in Study 1, but it may have an effect on the immediate cumulative structural priming. To explore this possibility, we modeled immediate cumulative structural priming including ‘other’ responses and with the following variables as fixed effects: (a) Age (3 vs. 4 vs. 6); (b) Prime Syntax (SVO vs. BA); and (c) Bilingualism (monolingual vs. bilingual). The results indicated that the main effects of Prime Syntax (*B* = 2.8383, *SD* = 0.3428, *z* = 8.279, *p* < 0.01) and Age (*B* = 0.2890, *SD* = 0.1344, *z* = 2.151, *p* = 0.0315) were significant, whereas the remaining effects were non-significant (*all p* > 0.05). The main effect of Prime Syntax shows that there was an overall priming effect with more BA responses after a blocked presentation of the BA primes than after the blocked presentation of the SVO primes. The main effect of Age suggests that there was at least one age group that produced more BA responses than the other age group(s). To reveal the age group(s) that may produce more BA responses than the other age group(s), a *post hoc* pairwise comparison model was performed using the emmeans function in R. The marginally significant contrast effect between 3-year-olds and 6-year-olds (*B* = −0.943, *SE* = 0.422, *z* = −2.235, *p* = 0.0654) indicate that 6-year-olds produced marginally more BA responses than their 3-year-old counterparts while the remaining contrasts among 3- and 4-year-olds, and 4- and 6-year-olds were not significant.

**FIGURE 3:**
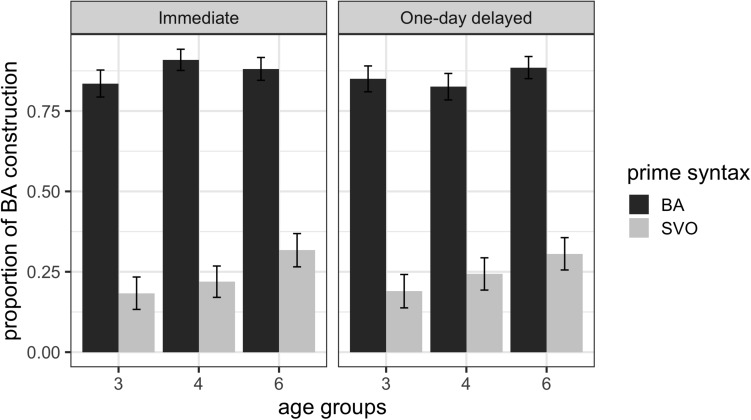
Mean proportions of *ba*-construction utterances with error bars representing standard errors of the means after SVO primes and BA primes across the three age groups in the immediate and 1-day delayed cumulative structural priming conditions.

We then investigated how this cumulative structural priming might change when children’s exposure to the primes and their production of the target animations were separated by 1-day. Figure 3 shows the mean proportions of the *ba*-construction utterances produced by children in the three age groups in the immediate cumulative structural priming and 1-day delayed cumulative structural priming conditions.

To explore the persistence of cumulative structural priming after a 1-day delay between the prime and target, we modeled the following variables as fixed effects: (a) Age (3 vs. 4 vs. 6); (b) Prime Syntax (SVO vs. BA); (c) Immediacy (immediate vs. 1-day delayed); and (d) Bilingualism (monolingual vs. bilingual) and treated participants and items as random effects. The results of this model are summarized in Table 5.

**TABLE 5:** Mixed logit model excluding ‘other’ responses: persistence of abstract cumulative structural priming.

**Mixed logit analysis results**				

**Predictor**	**Coefficient**	** Std. error**	**Wald Z**	***p*-value**
Intercept	0.5784	0.3741	1.546	0.122
Prime syntax	4.8735	0.5943	8.200	<0.001
Age	0.3475	0.2160	1.609	0.108
Immediacy	–0.1135	0.2457	–0.462	0.644
Bilingualism	–0.1064	0.3703	–0.287	0.774
Prime syntax × age	–0.1159	0.4218	–0.275	0.783
Prime syntax × immediacy	–0.2991	0.4873	–0.614	0.539
Age × immediacy	0.0470	0.2045	0.230	0.818
Prime syntax × bilingualism	–0.2912	0.7311	–0.398	0.690
Immediacy × bilingualism	–0.4652	0.6928	–0.671	0.502
Age × bilingualism	–0.0671	0.3115	–0.215	0.829
Prime syntax × age × immediacy	0.1412	0.4010	0.352	0.725
Prime syntax × age × bilingualism	–0.4856	0.6097	–0.796	0.426
Prime syntax × immediacy × bilingualism	–0.0054	1.3688	–0.004	0.997
Immediacy × age × bilingualism	0.0913	0.6311	0.145	0.885
Prime syntax × age × immediacy × bilingualism	0.4764	1.2343	0.386	0.700

The results indicate that there was a reliable (cumulative) structural priming effect as the Prime Syntax factor was significant (*p* < 0.001). They also show that neither the factors Age and Bilingualism nor the delay from the blocked presentation of the prime sentences to the target descriptions had an effect on structural priming (all *p* > 0.05). Again, the main effect of Prime Syntax shows that there was an overall priming effect with more BA responses after a blocked presentation of the BA primes than after the blocked presentation of the SVO primes. The theoretical *R*^2^_GLMM(_*_m_*_)_ of the model is 0.4587, and the theoretical *R*^2^_GLMM(_*_c),_* is 0.7485, suggesting that a moderate amount of the variability in BA responses can be explained by both fixed and random effects. A model that included the ‘other’ responses obtained the same overall results.

The finding that children’s cumulative structural priming effect is greater in magnitude than the structural priming effect and that it does not diminish after a 1-day delay between prime and target descriptions suggests that structural priming operates on an implicit learning mechanism that tracks the usage probabilities for the syntactic structures in a context-dependent way similar to adults (Kaschak et al., 2014). However, it is possible that the greater cumulative structural priming in Experiment 2 compared to Experiment 1 is due to increased exposure to a particular structure in terms of the number of primes. Specifically, children received four primes per structure in Experiment 1, but eight primes in one structure in Experiment 2. It is therefore not possible to fully distinguish between whether the increase in structural priming magnitude is due to cumulative structural priming or just hearing more instances of a particular prime structure. That said, Jaeger and Snider’s (2013) study 3 showed that when the number of the primes is controlled with one of the conditions alternately presenting the two types of primes and the other condition presenting the primes in two blocks, the blocked presentation of primes led to greater cumulative structural priming, which replicated Kaschak and colleagues’ findings (Kaschak, 2007; Kaschak et al., 2011). The lack of an age effect suggests that children in all age groups have abstract syntactic representations and track the use of the syntactic probabilities in the experiments, in a context-dependent manner (Kaschak et al., 2014).

The current results may also shed some light on the debate regarding the part of speech of *ba*, which was once thought to be a verb in the Tang dynasty from approximately 700 A.D., a controversial idea now because BA has lost most of its verb-hood between the time of the 18th century text *Rulin Waishi* and the 19th text *Ernu Yinxiong Zhuan* (Sun, 1997). Should *ba* be encoded as a verb among Mandarin-speaking preschoolers, we would expect a lexical boost effect. In addition, if *ba* triggered a lexical boost effect rather than a structural priming effect, the effect should not last into the next day (cf. Hartsuiker et al., 2008).

## General Discussion and Conclusion

Mandarin-speaking 3-, 4-, and 6-year-olds exhibited structural priming with an SVO-*ba* alternation after they comprehended the prime sentence only. These preschoolers exhibited a greater magnitude of cumulative structural priming than structural priming, which persisted after a 1-day delay. These results suggest young children as a group can exhibit reliable structural priming effects in this context. The greater cumulative structural priming compared to regular structural priming that persist for at least 1-day further, suggests that the exhibition of structural priming involves implicit learning.

### On Developmental Trajectory

Kidd (2012) argued that age played no role in children’s exhibition of structural priming and that the apparent age effects obtained could be attributed to individual differences in young children, such as vocabulary size and non-linguistic reasoning processes during testing. However, some studies (Rowland et al., 2012; Peter et al., 2015) on structural priming with the English dative alternation report an age effect, indicating that children’s exhibition of structural priming may depend on the particular syntactic alternation. Tomasello (2000) argued that developmental trajectories in terms of syntactic competence in young children may differ depending on the types of syntactic structure examined. This may be the case in the studies on structural priming in young children. The structural priming results of Study 1 align with the findings for the English active-passive alternation (Messenger et al., 2012), but not for the English dative alternation, in that they demonstrate that Mandarin-speaking preschoolers’ exhibition of structural priming remains stable across age groups without an observable developmental trajectory.

Developmental trajectories may also be affected by the task or analysis method. Comparisons of the studies by Messenger et al. (2012) and Hsu (2018) with the current study indicate that adults and older children performed substantially better than younger children in a forced-choice pointing paradigm, clearly indicating an age effect, whereas they did not exhibit this effect in the structural priming paradigm. Similarly, cumulative structural priming in Mandarin-speaking children was not affected by age when the ‘other’ responses were excluded. However, an age effect occurred when the ‘other’ responses were included in the analysis. This effect is most likely due to 3-year-olds producing more ‘other’ responses than their 6-year-old counterparts. Although the strength of structural priming effects remained stable across age groups, 3-year-olds’ production of relatively more ‘other’ responses might be due to non-linguistic reasoning factors in the younger group or their less-developed memories (Shimpi et al., 2007; Kidd, 2012), which might interfere with their use of developed abstract syntactic representations to track the usage of structures in the input received.

### On Structural Priming as Learning

Recent studies on structural priming suggest that a single prime is sufficient to induce structural priming in contexts that involve a comprehension-to-production paradigm, both in adults (Bock et al., 2007; Tooley and Bock, 2014) and children (Messenger et al., 2012; Rowland et al., 2012; Peter et al., 2015). Considering the earlier null results in comprehension-to-production paradigms (Shimpi et al., 2007), it is possible that more than one prime may facilitate structural priming in these contexts. The stronger cumulative compared to simple priming effect in the current study supports this idea. This suggests that both young children and adults (see a review in Jaeger and Snider, 2013) may track the usage probabilities of the syntactic structures when multiple utterances of a single structure serve as primes in the input. Thus, both children and adults employ the abstract syntactic representations in a context-dependent manner, and exhibit greater cumulative compared to regular structural priming effects.

The current study also found persistent cumulative structural priming in young children. The differences in the persistence of structural priming effects between children and adults may be due to the different types of alternations used across studies (Bock and Griffin, 2000). Persistent cumulative structural priming effects in adults have been found for the dative alternation (Kaschak et al., 2006, 2014), but not for the active-passive alternation (Messenger et al., 2012). To the best of our knowledge, it is mostly the active-passive alternation that has been examined in child language, and the passive structure is known to be difficult for children to comprehend. Young children’s difficulty in understanding the passive structure due to the reversal of the thematic role assignment (Messenger et al., 2012) may have an effect on their demonstrations of a persistent structural priming effect as Kidd (2012) has reported a significant decline of the cumulative structural priming effect in the immediate posttest block. Young English-speaking children may be unlikely to exhibit persistent cumulative structural priming effects beyond the immediate context without aids that enable them to overcome the difficulty of comprehending the passive structure (cf. a manipulation in Savage et al., 2006). Using the Mandarin-specific SVO-*ba* alternation, where both structures in the alternation share the same assignment of thematic roles (the first NP as the agent and the second NP as the patient), may allow children to overcome this difficulty and achieve long-lasting cumulative structural priming effects.

Even though adults can demonstrate a persistent cumulative structural priming effect with the dative alternation, children may be constrained by performance factors, such as memory, as Shimpi et al. (2007) have suggested. Gleaning from the data reported by Shimpi et al. (2007), it appears that young children seem to have more difficulty producing dative structures because their ‘other’ rates were numerically much higher than for the transitive counterparts. It is likely that some performance factors, such as memory, can prevent young children from producing dative structures. Such performance factors may have led to the null results of structural priming with the dative alternation from comprehension-to-production in English-speaking 3-year-olds, and may also prevent a reliable persistent cumulative structural priming effect.

Although the *ba*-construction is much less frequent than the SVO construction (Huang et al., 2013), their identical and canonical arrangement of agent and patient roles may enable children to perform similarly in comprehension (Hsu, 2018). The fact that the ‘other’ rates for structural priming with this alternation in young children are similar to those reported in studies on the English active-passive alternation in child structural priming suggests that young children have less difficulty producing transitive structures compared to dative structures. This may maximize children’s chances of tracking the usage of syntactic structures in this specific context, leading to a cumulative structural priming effect.

Above all, the current findings support the specific hypotheses that structural priming in Mandarin is derived from an abstract syntactic representation (Fisher, 2002; Naigles, 2002) that adapts itself in response to the input received (Bock and Griffin, 2000; Chang et al., 2006; Bock et al., 2007; Kaschak et al., 2014). These effects support the claim that young children can employ abstract syntactic representations beginning at the age of three in a language other than English.

## Ethics Statement

This study involving human participants has been approved by the author’s Institutional Review Board (IRB) in Taiwan (Research Ethics Committee, National Taiwan University and National Taiwan Normal University). The approval numbers are NTU-REC No. 201506ES028 and NTNU-REC No. 201805HS028. Informed written consent has been obtained from the participants for the adult participants and from the child participants’ parents.

## Author Contributions

The author confirms being the sole contributor of this work and has approved it for publication.

## Conflict of Interest

The author declares that the research was conducted in the absence of any commercial or financial relationships that could be construed as a potential conflict of interest.

## Appendix

### Target Animations for the SVO-ba Alternation

1. Laohu xiapao    -le xiaonanhai./Laohu ba xiaonanhai xiapao    -le.
  “A tiger scared a little boy (away).”
2. Maoxiong yabian    -le    houzi./Maoxiong ba houzi yabian    -le.
  “A panda pressed down and crushed a monkey.”
3. Xiaogou tuozou    -le    xiaobaitu./Xiaogou    ba    xiabaitu    tuozou    -le.
  “A dog pulled a rabbit away.”
4. Dayelan ganpao    -le    xiaozhu./Dayelan ba xiaozhu    ganpao    -le.
  “A wolf chased a pig and the pig ran away.”
5. Hema chueifei    -le maomi./Hema ba maomi chueifei    -le.
  “A hippo blew on a cat and the cat soared away.”
6. Houzi tuidao    -le    shizi./Houzi ba    shizi    tuidao    -le.
  “A monkey pushed a lion and the lion turned and then fell down.”
7. Xiong taiqi    -le    xiniu./Xiong ba xiniu taiqi    -le.
  “A bear lifted a rhino.”
8. Laoyeye    dashang    -le    yan./Laoyeye    ba    yan    dashang    -le.
  “An old man hit and hurt the sheep.”
